# Putting the BMC into psychology publishing

**DOI:** 10.1186/2050-7283-1-1

**Published:** 2013-02-27

**Authors:** Gordon Harold

**Affiliations:** Developmental Psychopathology, University of Leicester, Leicester, UK

This editorial celebrates the launch of *BMC Psychology*[1], the newest addition to the *BMC series*[2] journal group. *BMC Psychology* is an open access, peer-reviewed journal that considers manuscripts on all aspects of psychology, human behavior and the mind, including developmental, clinical, cognitive, experimental, social, evolutionary and educational psychology, as well as personality and individual differences. The journal welcomes quantitative and qualitative research methods, including animal studies. It is journal policy to publish work deemed by peer reviewers to be a coherent and sound addition to scientific knowledge and to put less emphasis on interest levels, provided sthat the research constitutes a useful contribution to the field. In this editorial, Gordon Harold, Section Editor for Developmental psychopathology, discusses why this journal will be a valuable addition to the psychology milieu, and the aims of the journal.

Welcome to *BMC Psychology*! An exciting and innovative new publication outlet aimed at the dissemination of cutting-edge research relevant to the field of psychological science and complementary disciplines.

Psychology as a discipline has a recognised and well-documented history, with many specialist academic journals in existence that capture the breadth of topics and specialist areas relevant to what remains a relatively young field of scientific enquiry. Since the inception of psychology as a scientific discipline at the turn of the 20th century, psychology has passed through many transitions on its way to becoming a recognised scientific discipline with relevance to all aspects of human behaviour and development. Uniquely, rather than evolve into a subject defined by very strict disciplinary-based boundaries across the course of its developmental history, psychology as a science benefits from a set of sub-disciplines that each offer specificity in terms of primary scientific enquiry, while also offering synergies across areas within the field of psychology as well as disciplines that fall outside the core boundaries of psychology (e.g. medicine, law, genetics, social work). Indeed, psychology is, unlike many other scientific disciplines, a true interdisciplinary science. The strength of the discipline relative to other areas of scientific enquiry is that where links can be established with other disciplines, psychology as a subject area has the flexibility to adapt and accommodate new avenues of scientific potential, enquiry and application without compromising its core methodological and theoretical foundations. This potential is recognised and captured through the ethos and publication orientation of *BMC Psychology*.

One of the key contemporary challenges for most areas of modern science is to move from what may be described as distal-level enquiry to proximal-level application. In other words, there is an increasing demand and need for the product of scientific enquiry to move from the static pages of remote scientific journals to real world applications, public awareness and impact through the translation and dissemination of such science. This poses both challenge and great opportunity for the field of psychology. Through the development of *BMC Psychology*, a forum is offered that encapsulates the interface between rigorous science, the potential for practical and clinical application, as well as core knowledge development and dissemination relating to all aspects of human behaviour and development, including animal studies.

As Section Editor for the Developmental Psychopathology section of the journal, I am very keen to promote the scientific objectives of this realm of psychological science in concert with core objectives of the journal. The field of developmental psychopathology constitutes the scientific study of normal and abnormal adaptation and psychological development across the lifespan. The subject area takes a rigorous research orientation to examination of primary theoretical questions, with a bottom-line practice and policy orientation to primary scientific outputs. To have this area of psychological science represented “under one umbrella” in company with an expansive set of complementary subject areas and specialist disciplines across the field of psychology emphasises the forward thinking, ambition and novelty of this newly established journal – a journal of relevance to students, established researchers, practitioners, policy makers and educators alike.

As the journal profile outlines, *BMC Psychology* is an open access, peer-reviewed journal that considers manuscripts on all aspects of psychology and human behaviour, including developmental, clinical, cognitive, experimental, social, evolutionary, and educational psychology, as well as personality and individual differences. The journal welcomes submission of articles that employ quantitative, qualitative and mixed methods approaches, as well as studies that utilise non-human subjects (e.g., animal studies). A common denominator across all areas and topics of interest to *BMC Psychology*, however, is *rigour* and *relevance*; both in terms of the scientific quality of studies published as well as the potential for public, practice and policy impacts of study findings where relevant.

One of the key distinguishing factors between *BMC Psychology* and other psychology journals is that *BMC Psychology* will be open peer review. "Open peer review" [3] means that, firstly, the reviewers' names are included on the peer review reports, and secondly that, if the manuscript is published, the reports are made available online along with the different versions of the manuscript and the responses of the authors to each review. The published article will provide a link to this 'pre-publication history'.

All of the medical journals within the *BMC Series* are open peer review, as the publishers consider it supports and enhances transparent and impartial publishing. It also encourages reviewers to provide a thorough and constructive review. In a 2006 trial conducted by *Nature*[4] one the key reasons for choosing open peer review was reviewer accountability which is likely to lead to higher quality reports and to decrease any potential bias, and to encourage better communication between authors and reviewers.

Being part of a broader family of journals comprised by the *BMC Series* also offers some additional benefits. Not only can the journal production team and editorial boards find a manuscript the best possible home within the series according to its scope, but with a simple transfer, outstanding and broad interest pieces that fit the criteria of *BMC Medicine*[5] may be offered publication in this flagship journal. In addition, if the manuscript does not quite meet the criteria for *BMC Psychology*, but is still sound science, then we can offer a transfer to *BMC Research Notes*[6] – in all these cases, any peer reviews can be transferred along with the manuscript, meaning that the peer review process does not have to be initiated again. The editorial team may also highlight strong articles, when published, on the main BioMed Central homepage [7], and encourage comments and discussion on articles – not to mention blog posts and distribution with other social media means. All in all, a comprehensive package of dissemination and distribution possibilities!

We welcome submission of your work and the contributions you can make to help further advance the field of psychological science through the pages of *BMC Psychology*!
